# Tetra­aqua­bis[2-(2,4-dichloro­phen­oxy)acetato]nickel(II)

**DOI:** 10.1107/S1600536809033662

**Published:** 2009-08-29

**Authors:** Wu Chen, Ji-Wen Yuan, Lei Lei, Qing-Fu Zeng

**Affiliations:** a Engineering Research Center for Clean Production of Textile Dyeing and Printing, Ministry of Education, Wuhan 430073, People’s Republic of China

## Abstract

In the title complex, [Ni(C_8_H_5_Cl_2_O_3_)_2_(H_2_O)_4_], the Ni^II^ atom (site symmetry 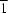) adopts a slightly distorted NiO_6_ octa­hedral coordination. An intra­molecular O—H⋯O hydrogen bond helps to establish the conformation. In the crystal, further O—H⋯O hydrogen bonds link the mol­ecules.

## Related literature

For background, see: Cheng *et al.* (2006 ▶). For reference structural data, see: Allen *et al.* (1987 ▶).
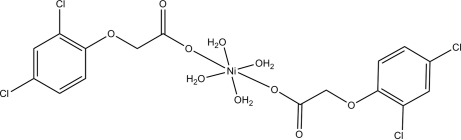


## Experimental

### 

#### Crystal data

[Ni(C_8_H_5_Cl_2_O_3_)_2_(H_2_O)_4_]
*M* *_r_* = 570.81
Monoclinic, 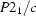

*a* = 16.860 (3) Å
*b* = 8.1370 (16) Å
*c* = 8.3010 (17) Å
β = 95.87 (3)°
*V* = 1132.8 (4) Å^3^
*Z* = 2
Mo *K*α radiation
μ = 1.38 mm^−1^
*T* = 293 K
0.30 × 0.20 × 0.10 mm

#### Data collection

Enraf–Nonius CAD-4 diffractometer
Absorption correction: ψ scan (North *et al.*, 1968 ▶) *T*_min_ = 0.683, *T*_max_ = 0.875
2134 measured reflections
1976 independent reflections
1596 reflections with *I* > 2σ(*I*)
*R*_int_ = 0.017
200 standard reflections every 3 reflections intensity decay: 1%

#### Refinement

*R*[*F* ^2^ > 2σ(*F* ^2^)] = 0.071
*wR*(*F* ^2^) = 0.214
*S* = 1.14
1976 reflections
154 parameters
6 restraints
H atoms treated by a mixture of independent and constrained refinement
Δρ_max_ = 0.86 e Å^−3^
Δρ_min_ = −1.97 e Å^−3^

### 

Data collection: *CAD-4 Software* (Enraf–Nonius, 1989 ▶); cell refinement: *CAD-4 Software*; data reduction: *XCAD4* (Harms & Wocadlo, 1995 ▶); program(s) used to solve structure: *SHELXS97* (Sheldrick, 2008 ▶); program(s) used to refine structure: *SHELXL97* (Sheldrick, 2008 ▶); molecular graphics: *SHELXTL* (Sheldrick, 2008 ▶); software used to prepare material for publication: *SHELXTL*.

## Supplementary Material

Crystal structure: contains datablocks global, I. DOI: 10.1107/S1600536809033662/hb5064sup1.cif

Structure factors: contains datablocks I. DOI: 10.1107/S1600536809033662/hb5064Isup2.hkl

Additional supplementary materials:  crystallographic information; 3D view; checkCIF report

## Figures and Tables

**Table 1:** Selected bond lengths (Å)

Ni1—O3	2.085 (5)
Ni1—O4	2.126 (4)
Ni1—O1	2.130 (4)

**Table 2:** Hydrogen-bond geometry (Å, °)

*D*—H⋯*A*	*D*—H	H⋯*A*	*D*⋯*A*	*D*—H⋯*A*
O1—H1*A*⋯O2^i^	0.84 (5)	2.05 (7)	2.723 (7)	136 (8)
O1—H1*B*⋯O2	0.84 (3)	1.82 (5)	2.619 (7)	157 (7)
O3—H3*A*⋯O1^i^	0.85 (6)	2.44 (7)	3.217 (7)	153 (7)
O3—H3*B*⋯O6^ii^	0.846 (16)	2.34 (6)	2.980 (7)	133 (8)

Symmetry codes: (i) 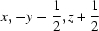; (ii) 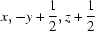.

**Fig. 1.**
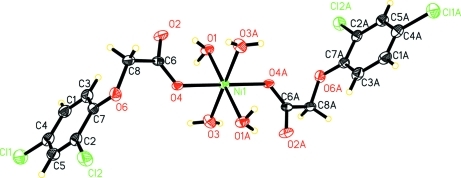
The molecular structure of (I) showing 30% probability displacement ellipsoids. Atoms with the suffix A are generated by the symmetry operation (1–x, –y, 1–z).

## supplementary crystallographic information

### Comment

There has been much research interest in acid metal complexes due to their molecular architectures and biological activities (e.g. Cheng *et al.*, 2006). In this work, we report here the crystal structure of the title compound, (I). In (I), all bond lengths are within normal ranges (Allen *et al.*, 1987) (Fig. 1). The Ni^II^ atom is six-coordinated by two O atoms from the 2-(2,4-dichlorophenoxy)acetate and four O atoms from the water molecules, forming a slightly distorted octahedral coordination.

### Experimental

A mixture of 2-(2,4-dichlorophenoxy)acetic acid (440 mg, 2 mmol) and NiCl2.6H2O (1 mmol, 236 mg) in methanol (10 ml) was stirred for 3 h. After keeping the filtrate in air for 7 d, green blocks of (I) were formed.

### Refinement

The water H atoms were located in a difference map and their positions were refined with the restraint O—H = 0.83 (1)Å. The other H atoms were positioned geometrically (C—H = 0.93–0.97 Å) and refined as riding, with *U*_iso_(H) = 1.2*U*_eq_(C).

### Crystal data

**Table:**

[Ni(C_8_H_5_Cl_2_O_3_)_2_(H_2_O)_4_]	*F*(000) = 580
*M**_r_* = 570.81	*D*_x_ = 1.673 Mg m^−^^3^
Monoclinic, *P*2_1_/*c*	Mo *K*α radiation, λ = 0.71073 Å
Hall symbol: -P 2ybc	Cell parameters from 25 reflections
*a* = 16.860 (3) Å	θ = 9–12°
*b* = 8.1370 (16) Å	µ = 1.38 mm^−^^1^
*c* = 8.3010 (17) Å	*T* = 293 K
β = 95.87 (3)°	Block, green
*V* = 1132.8 (4) Å^3^	0.30 × 0.20 × 0.10 mm
*Z* = 2	

### Data collection

**Table:**

Enraf–Nonius CAD-4 diffractometer	1596 reflections with *I* > 2σ(*I*)
Radiation source: fine-focus sealed tube	*R*_int_ = 0.017
graphite	θ_max_ = 25.2°, θ_min_ = 1.2°
ω/2θ scans	*h* = −20→20
Absorption correction: ψ scan (North *et al.*, 1968)	*k* = −9→0
*T*_min_ = 0.683, *T*_max_ = 0.875	*l* = 0→9
2134 measured reflections	200 standard reflections every 3 reflections
1976 independent reflections	intensity decay: 1%

### Refinement

**Table:**

Refinement on *F*^2^	Primary atom site location: structure-invariant direct methods
Least-squares matrix: full	Secondary atom site location: difference Fourier map
*R*[*F*^2^ > 2σ(*F*^2^)] = 0.071	Hydrogen site location: inferred from neighbouring sites
*wR*(*F*^2^) = 0.214	H atoms treated by a mixture of independent and constrained refinement
*S* = 1.14	*w* = 1/[σ^2^(*F*_o_^2^) + (0.1181*P*)^2^ + 3.965*P*] where *P* = (*F*_o_^2^ + 2*F*_c_^2^)/3
1976 reflections	(Δ/σ)_max_ = 0.001
154 parameters	Δρ_max_ = 0.86 e Å^−^^3^
6 restraints	Δρ_min_ = −1.97 e Å^−^^3^

### Special details

**Table:**

Geometry. All e.s.d.'s (except the e.s.d. in the dihedral angle between two l.s. planes) are estimated using the full covariance matrix. The cell e.s.d.'s are taken into account individually in the estimation of e.s.d.'s in distances, angles and torsion angles; correlations between e.s.d.'s in cell parameters are only used when they are defined by crystal symmetry. An approximate (isotropic) treatment of cell e.s.d.'s is used for estimating e.s.d.'s involving l.s. planes.
Refinement. Refinement of *F*^2^ against ALL reflections. The weighted *R*-factor *wR* and goodness of fit *S* are based on *F*^2^, conventional *R*-factors *R* are based on *F*, with *F* set to zero for negative *F*^2^. The threshold expression of *F*^2^ > σ(*F*^2^) is used only for calculating *R*-factors(gt) *etc*. and is not relevant to the choice of reflections for refinement. *R*-factors based on *F*^2^ are statistically about twice as large as those based on *F*, and *R*- factors based on ALL data will be even larger.

### Fractional atomic coordinates and isotropic or equivalent isotropic displacement parameters (Å^2^)

**Table:**

	*x*	*y*	*z*	*U*_iso_*/*U*_eq_	
C1	0.1103 (4)	0.1141 (9)	0.3299 (9)	0.0487 (17)	
H1	0.0872	0.0160	0.3585	0.058*	
C2	0.1780 (4)	0.4056 (8)	0.2486 (8)	0.0363 (14)	
C3	0.1822 (4)	0.1110 (8)	0.2642 (9)	0.0454 (16)	
H3	0.2076	0.0113	0.2504	0.054*	
C4	0.0724 (4)	0.2598 (9)	0.3534 (9)	0.0458 (16)	
C5	0.1072 (4)	0.4079 (8)	0.3131 (8)	0.0431 (15)	
H5	0.0821	0.5072	0.3303	0.052*	
C6	0.3693 (4)	0.0240 (7)	0.2247 (7)	0.0296 (12)	
C7	0.2173 (4)	0.2580 (7)	0.2183 (8)	0.0345 (13)	
C8	0.3201 (4)	0.1188 (8)	0.0942 (7)	0.0388 (15)	
H8A	0.3533	0.1447	0.0088	0.047*	
H8B	0.2770	0.0489	0.0479	0.047*	
Cl1	−0.01949 (12)	0.2621 (3)	0.4316 (3)	0.0626 (6)	
Cl2	0.22352 (11)	0.5880 (2)	0.1988 (3)	0.0557 (6)	
H1A	0.418 (3)	−0.233 (12)	0.523 (6)	0.067*	
H3A	0.452 (4)	−0.018 (10)	0.785 (6)	0.067*	
H1B	0.436 (4)	−0.226 (11)	0.366 (3)	0.067*	
H3B	0.3858 (7)	0.030 (11)	0.687 (9)	0.067*	
Ni1	0.5000	0.0000	0.5000	0.0266 (4)	
O1	0.4550 (3)	−0.2430 (5)	0.4624 (6)	0.0406 (11)	
O2	0.3738 (3)	−0.1289 (5)	0.2003 (5)	0.0433 (11)	
O3	0.4362 (3)	0.0347 (7)	0.6996 (6)	0.0503 (12)	
O4	0.4045 (2)	0.0994 (5)	0.3429 (5)	0.0319 (9)	
O6	0.2866 (3)	0.2687 (6)	0.1489 (6)	0.0420 (11)	

### Atomic displacement parameters (Å^2^)

**Table:**

	*U*^11^	*U*^22^	*U*^33^	*U*^12^	*U*^13^	*U*^23^
C1	0.053 (4)	0.039 (4)	0.052 (4)	−0.005 (3)	−0.005 (3)	−0.005 (3)
C2	0.038 (3)	0.029 (3)	0.041 (3)	−0.008 (3)	−0.002 (3)	0.005 (3)
C3	0.048 (4)	0.027 (3)	0.059 (4)	−0.002 (3)	−0.009 (3)	0.004 (3)
C4	0.049 (4)	0.046 (4)	0.040 (4)	−0.006 (3)	−0.006 (3)	0.002 (3)
C5	0.049 (4)	0.032 (3)	0.047 (4)	0.000 (3)	0.000 (3)	0.000 (3)
C6	0.039 (3)	0.018 (3)	0.032 (3)	0.001 (2)	0.006 (2)	−0.002 (2)
C7	0.037 (3)	0.029 (3)	0.036 (3)	0.004 (2)	−0.004 (2)	0.000 (2)
C8	0.046 (4)	0.035 (3)	0.034 (3)	0.006 (3)	−0.001 (3)	−0.009 (3)
Cl1	0.0543 (11)	0.0675 (13)	0.0680 (13)	−0.0061 (9)	0.0147 (9)	−0.0037 (10)
Cl2	0.0552 (11)	0.0276 (8)	0.0841 (14)	0.0013 (7)	0.0062 (9)	0.0059 (8)
Ni1	0.0369 (6)	0.0154 (5)	0.0270 (6)	0.0015 (4)	0.0017 (4)	0.0023 (4)
O1	0.058 (3)	0.028 (2)	0.035 (2)	0.000 (2)	−0.001 (2)	−0.0015 (19)
O2	0.066 (3)	0.031 (2)	0.032 (2)	−0.001 (2)	−0.003 (2)	−0.0056 (18)
O3	0.048 (3)	0.054 (3)	0.050 (3)	0.009 (2)	0.013 (2)	0.007 (2)
O4	0.043 (2)	0.0205 (19)	0.030 (2)	0.0092 (17)	−0.0054 (17)	−0.0048 (17)
O6	0.037 (2)	0.032 (2)	0.056 (3)	0.0010 (18)	−0.002 (2)	0.004 (2)

### Geometric parameters (Å, °)

**Table:**

C1—C4	1.369 (10)	C7—O6	1.358 (8)
C1—C3	1.381 (11)	C8—O6	1.437 (7)
C1—H1	0.9300	C8—H8A	0.9700
C2—C5	1.358 (10)	C8—H8B	0.9700
C2—C7	1.407 (9)	Ni1—O3	2.085 (5)
C2—Cl2	1.740 (6)	Ni1—O3^i^	2.085 (5)
C3—C7	1.403 (9)	Ni1—O4	2.126 (4)
C3—H3	0.9300	Ni1—O4^i^	2.126 (4)
C4—C5	1.396 (10)	Ni1—O1^i^	2.130 (4)
C4—Cl1	1.741 (8)	Ni1—O1	2.130 (4)
C5—H5	0.9300	O1—H1A	0.841 (10)
C6—O4	1.253 (7)	O1—H1B	0.840 (10)
C6—O2	1.265 (7)	O3—H3A	0.844 (10)
C6—C8	1.508 (8)	O3—H3B	0.847 (10)
			
C4—C1—C3	120.9 (7)	C6—C8—H8B	108.7
C4—C1—H1	119.6	H8A—C8—H8B	107.6
C3—C1—H1	119.6	O3—Ni1—O3^i^	180.0
C5—C2—C7	122.1 (6)	O3—Ni1—O4	90.93 (19)
C5—C2—Cl2	120.6 (5)	O3^i^—Ni1—O4	89.07 (18)
C7—C2—Cl2	117.3 (5)	O3—Ni1—O4^i^	89.07 (18)
C1—C3—C7	120.2 (6)	O3^i^—Ni1—O4^i^	90.93 (18)
C1—C3—H3	119.9	O4—Ni1—O4^i^	180.0
C7—C3—H3	119.9	O3—Ni1—O1^i^	87.9 (2)
C1—C4—C5	120.0 (7)	O3^i^—Ni1—O1^i^	92.1 (2)
C1—C4—Cl1	120.5 (6)	O4—Ni1—O1^i^	88.46 (16)
C5—C4—Cl1	119.5 (6)	O4^i^—Ni1—O1^i^	91.54 (16)
C2—C5—C4	119.4 (6)	O3—Ni1—O1	92.1 (2)
C2—C5—H5	120.3	O3^i^—Ni1—O1	87.9 (2)
C4—C5—H5	120.3	O4—Ni1—O1	91.54 (16)
O4—C6—O2	125.2 (5)	O4^i^—Ni1—O1	88.46 (16)
O4—C6—C8	119.6 (5)	O1^i^—Ni1—O1	180.0
O2—C6—C8	115.2 (5)	Ni1—O1—H1A	96 (7)
O6—C7—C3	125.1 (6)	Ni1—O1—H1B	94 (6)
O6—C7—C2	117.5 (5)	H1A—O1—H1B	108.9 (18)
C3—C7—C2	117.4 (6)	Ni1—O3—H3A	117 (6)
O6—C8—C6	114.4 (5)	Ni1—O3—H3B	119 (6)
O6—C8—H8A	108.7	H3A—O3—H3B	108.4 (18)
C6—C8—H8A	108.7	C6—O4—Ni1	124.2 (4)
O6—C8—H8B	108.7	C7—O6—C8	117.5 (5)
			
C4—C1—C3—C7	−0.9 (11)	O4—C6—C8—O6	−28.4 (8)
C3—C1—C4—C5	−1.0 (11)	O2—C6—C8—O6	154.2 (6)
C3—C1—C4—Cl1	178.4 (5)	O2—C6—O4—Ni1	14.9 (9)
C7—C2—C5—C4	1.0 (10)	C8—C6—O4—Ni1	−162.2 (4)
Cl2—C2—C5—C4	−179.3 (5)	O3—Ni1—O4—C6	−122.0 (5)
C1—C4—C5—C2	0.9 (10)	O3^i^—Ni1—O4—C6	58.0 (5)
Cl1—C4—C5—C2	−178.5 (5)	O4^i^—Ni1—O4—C6	76 (100)
C1—C3—C7—O6	−178.2 (6)	O1^i^—Ni1—O4—C6	150.1 (5)
C1—C3—C7—C2	2.7 (10)	O1—Ni1—O4—C6	−29.9 (5)
C5—C2—C7—O6	178.0 (6)	C3—C7—O6—C8	10.6 (9)
Cl2—C2—C7—O6	−1.6 (8)	C2—C7—O6—C8	−170.3 (5)
C5—C2—C7—C3	−2.8 (10)	C6—C8—O6—C7	−83.3 (7)
Cl2—C2—C7—C3	177.5 (5)		

Symmetry codes: (i) −*x*+1, −*y*, −*z*+1.

### Hydrogen-bond geometry (Å, °)

**Table:**

*D*—H···*A*	*D*—H	H···*A*	*D*···*A*	*D*—H···*A*
O1—H1A···O2^ii^	0.84 (5)	2.05 (7)	2.723 (7)	136 (8)
O1—H1B···O2	0.84 (3)	1.82 (5)	2.619 (7)	157 (7)
O3—H3A···O1^ii^	0.85 (6)	2.44 (7)	3.217 (7)	153 (7)
O3—H3B···O6^iii^	0.85 (2)	2.34 (6)	2.980 (7)	133 (8)

Symmetry codes: (ii) *x*, −*y*−1/2, *z*+1/2; (iii) *x*, −*y*+1/2, *z*+1/2.
